# The immunomodulatory role of tumor Syndecan-1 (CD138) on *ex vivo* tumor microenvironmental CD4^+^ T cell polarization in inflammatory and non-inflammatory breast cancer patients

**DOI:** 10.1371/journal.pone.0217550

**Published:** 2019-05-30

**Authors:** Moshira Ezzat Saleh, Ramy Gadalla, Hebatallah Hassan, Ahmed Afifi, Martin Götte, Mohamed El-Shinawi, Mona Mostafa Mohamed, Sherif Abdelaziz Ibrahim

**Affiliations:** 1 Department of Zoology, Faculty of Science, Cairo University, Giza, Egypt; 2 Department of Gynecology and Obstetrics, Münster University Hospital, Münster, Germany; 3 Department of General Surgery, Faculty of Medicine, Ain Shams University, Cairo, Egypt; University of Insubria, ITALY

## Abstract

Herein, we aimed to identify the immunomodulatory role of tumor Syndecan-1 (CD138) in the polarization of CD4^+^ T helper (Th) subsets isolated from the tumor microenvironment of inflammatory breast cancer (IBC) and non-IBC patients. Lymphocytes and mononuclear cells isolated from the axillary tributaries of non-IBC and IBC patients during modified radical mastectomy were either stimulated with the secretome as indirect co-culture or directly co-cultured with control and Syndecan-1-silenced SUM-149 IBC cells. In addition, peripheral blood mononuclear cells (PBMCs) of normal subjects were used for the direct co-culture. Employing flow cytometry, we analyzed the expression of the intracellular IFN-γ, IL-4, IL-17, and Foxp3 markers as readout for basal and co-cultured Th_1_, Th_2_, Th_17_, and T_reg_ CD4^+^ subsets, respectively. Our data revealed that IBC displayed a lower basal frequency of Th_1_ and Th_2_ subsets than non-IBC. Syndecan-1-silenced SUM-149 cells significantly upregulated only T_reg_ subset polarization of normal subjects relative to controls. However, Syndecan-1 silencing significantly enhanced the polarization of Th_17_ and T_reg_ subsets of non-IBC under both direct and indirect conditions and induced only Th_1_ subset polarization under indirect conditions compared to control. Interestingly, qPCR revealed that there was a negative correlation between Syndecan-1 and each of IL-4, IL-17, and Foxp3 mRNA expression in carcinoma tissues of IBC and that the correlation was reversed in non-IBC. Mechanistically, Syndecan-1 knockdown in SUM-149 cells promoted Th_17_ cell expansion via upregulation of IL-23 and the Notch ligand DLL4. Overall, this study indicates a low frequency of the circulating antitumor Th_1_ subset in IBC and suggests that tumor Syndecan-1 silencing enhances ex vivo polarization of CD4^+^ Th_17_ and T_reg_ cells of non-IBC, whereby Th_17_ polarization is possibly mediated via upregulation of IL-23 and DLL4. These findings suggest the immunoregulatory role of tumor Syndecan-1 expression in Th cell polarization that may have therapeutic implications for breast cancer.

## Introduction

Female breast cancer is the most broadly diagnosed cancer heading the list of life-threatening cancers in women all over the world and in Egypt [1, 2]. Inflammatory breast cancer (IBC) is a deadly aggressive form of breast cancer that is featured by enrichment of cancer stemness, rapid invasion into the dermal lymphatic vasculature, increasing metastasis, and low survival rate in comparison to non-IBC [3, 4]. One of the mechanistic clues for the clinical and pathological features of IBC are the components of the tumor microenvironment that can crosstalk with IBC cells either directly through cell-cell physical interactions [5] or indirectly via soluble paracrine mediators [6] to maintain tumor growth and escape immunosurveillance [7, 8].

In a previous study, we have detected a chronic inflammatory breast cancer microenvironment enriched with a high frequency of T helper cells (CD4^+^ T cells) that are considered to be important immune players within the tumor microenvironment [9]. CD4^+^ T cells can be polarized into paradoxical subsets; Th_1_, Th_2_, Th_17_, and T_reg_ in response to various signaling mediators [10]. The Th_1_ subset typically produces IFN-γ to enhance the cytotoxic activity of tumor-specific CD8^+^ T cells [11]. The Th_2_ subset produces the classical anti-inflammatory cytokines IL-4, IL-13, and IL-5, to activate the M_2_ macrophage protumor phenotype and increase metastasis potential of breast carcinoma [12]. The Th_17_ subset primarily produces IL-17A to promote proliferation, metastasis, and drug resistance of breast carcinoma [13, 14]. Finally, the Foxp3^+^ regulatory T cells (T_reg_) that create an immunosuppressive tumor microenvironment, allowing escape from immunosurveillance, and consequently, enhancing breast carcinoma progression [15, 16]. CD4^+^ T cells are highly plastic cells that can be inter-converted between different subsets according to cytokines and chemokines milieu of the environment [17].

One of the key molecules that are implicated in the modulation of inflammatory cytokines and chemokines, as well as the progression of breast carcinoma, is the heparan sulfate (HS) proteoglycan Syndecan-1 (Sdc-1; CD138) [3, 18]. In breast carcinoma, the differential expression and molecular functions have been assigned to Sdc-1 in the context of its stromal/cancer cell expression and/or its membrane-bound/shedding forms [19–21]. Recently, we have demonstrated that relative to non-IBC, carcinoma tissue of IBC confers a higher expression of Sdc-1 mRNA and protein that is associated with the expression of Notch as well as the unique cancer stemness phenotype of IBC [3]. On the other side, various studies provide evidence that ligand engagement with Notch receptor on the surface of Th cells can orchestrate the differentiation of Th subsets through regulation of their genetic signature [22]. Furthermore, Sdc-1 can inhibit T cell driven inflammation through sequestration of T cell-specific CC chemokines in a HS-dependent mechanism [23–25]. Accordingly, Sdc-1 depletion has been shown to modulate the expression of IL-17 and IFN-γ in natural killer T (NKT) cells during thymic development [26]. All these studies pinpoint an important role for Sdc-1 in reprogramming the soluble mediators that consequently shape the cellular composition of the inflammatory environment. Therefore, this prompted us to investigate the frequency of different T helper subsets drained from the tumor microenvironment of non-IBC and IBC through axillary tributaries. Moreover, we sought to unravel the potential unexplored immunomodulatory role played by Sdc-1 in modulating the polarization of CD4^+^ T cells isolated from non-IBC and IBC patients.

## Materials and methods

### Cell culture

The human inflammatory breast cancer (IBC) cell line SUM-149 (a kind gift from Dr. Bonnie Sloane, Wayne State University, Detroit, MI, USA) was maintained in HAM’s-F12 medium containing 5% fetal calf serum (FCS), 1% glutamine and 1% penicillin/streptomycin in a humidified atmosphere of 5% CO_2_ at 37 °C. All cell culture supplies were purchased from Lonza (MD, USA), unless otherwise stated.

### siRNA-mediated silencing of Sdc-1 expression

A total of 3.5 x 10^5^ SUM-149 cells were subjected to Sdc-1 silencing as we previously described [3]. Successful knockdown was confirmed by quantitative PCR (qPCR) and flow cytometry [27, 28].

### Preparation of media conditioned by the secretome of control and Sdc-1-silenced SUM-149 cells

For preparation of conditioned media (CM), 48 h post transfection control and Sdc-1-silenced SUM-149 cells were cultivated in serum free HAM’s-F12 media in a humidified atmosphere of 5% CO_2_ at 37°C for 24 hours. CM was collected, centrifuged at 1500 rpm for 5 min to remove cell debris and stored at −80°C for stimulation experiments.

### Patient samples

This study was approved by the research ethics committee of Ain Shams University, Cairo, Egypt (IRB#00006379), and has been conducted in accordance with the ethical standards of the 1964 Helsinki declaration. A written informed consent to participate in this study and to publish data was obtained from 55 female patients who were diagnosed with breast cancer and received primary surgery at breast clinic of Ain Shams University. Patients were divided into 2 groups: non-IBC (n = 34) and IBC (n = 21). During modified radical mastectomy, carcinoma tissues and 10 mL blood drained from tumor microenvironment to the venous circulation of the breast through axillary tributaries was collected in EDTA tubes as we described previously [9]. Normal breast tissues were collected during reduction mammoplatsty from healthy volunteers.

### Immunophenotyping of axillary CD4^+^ T cell subsets of non-IBC and IBC patients

Blood mononuclear cells were separated from axillary tributaries by Ficoll-hypaque density gradient according to manufacturer`s instructions. Briefly, whole blood was diluted with equal volume phosphate buffered saline (PBS) before Ficoll density gradient centrifugation. The buffy coat containing the mononuclear cells was harvested and red blood cells (RBCs) were lysed by incubation in ammonium chloride potassium (ACK) lysing buffer. Afterwards, the mononuclear cells were washed twice with cold PBS and monocytes were depleted by adherence to a plastic surface at 37°C for 2 hours [29], whereas the non-adherent lymphocytes were isolated and resuspended as 1 x 10^6^ cells/ml RPMI 1640 supplemented with 10% FCS, 1% glutamine, and 1% penicillin/ streptomycin for immunophenotyping by flow cytometry.

### Stimulation of T-lymphocytes by the secretome of control and Sdc-1-silenced SUM-149 cells

To elucidate the effect of tumor Sdc-1 expression on the polarization of CD4^+^ T cells, we activated the isolated lymphocytes with plate bound anti-CD3 (10 μg/ml; clone: MEM-57), and soluble anti-CD28 (0.5 μg/ml; clone: 15E8) (Immunotools, Friesoythe, Germany), in complete RPMI 1640 medium containing 30% CM of control and Sdc-1-silenced SUM-149 cells for 96 h. The optimal concentration of CM was selected based on our pilot experiments.

### Direct co-culture of mononuclear cells with control and Sdc-1-silenced SUM-149 cells

To investigate the immunoregulatory role of tumor Sdc-1 on polarization of CD4^+^ T cells under physical contact with cancer cells, we established a direct co-culture model of control and Sdc-1-silenced SUM-149 cells and mononuclear cells isolated from non-IBC patients or normal volunteers. 48 hours post transfection, the culture medium was carefully aspirated and fresh mononuclear cells were directly added into SUM-149 cells in a ratio of 5:1, respectively. The cells were cultured in 1:1 complete growth media of both RPMI 1640 and HAM’s-F12, and incubated in a humidified atmosphere of 5% CO_2_ at 37°C for 48 h.

### Flow cytometry

For assessment of intracellular cytokine expression in CD4^+^ T cells, the lymphocytes were incubated with 10 μg/mL brefeldin A (Sigma-Aldrich, St. Louis, MO, USA). After 6 hours, cells were harvested and stained with anti-CD4-FITC (eBioscience, Inc., San Diego, CA, USA) for 30 min at room temperature, followed by fixation and permeabilization using the Cytofix/Cytoperm Kit (BD Biosciences, San Diego, CA, USA) according to the manufacturer’s instructions. The intracellular cytokine staining was performed using the following monoclonal antibodies: IFN-γ-PE, IL-4-PEcy7, IL-17-PE, and Foxp3-PEcy7 (eBioscience, Inc., San Diego, CA, USA). Stained cells were analyzed by a cube-8 flow cytometer (Sysmex/Partec, Münster, Germany). Gates were applied to define the populations of interest, and the analysis was carried out using FCS Express 4 (De Novo Software, CA, USA). Isotype controls were used as negative controls.

### Quantitative real-time PCR

Total RNA from the breast carcinoma tissue (non-IBC and IBC), control and Sdc-1 siRNA transfected SUM-149 cells was extracted using the RNA Purification Kit GeneJET (Thermo scientific, ON, Canada) and reverse transcribed into complementary DNA (cDNA) using High-Capacity cDNA Reverse Transcription Kit (Thermo scientific, ON, Canada). qPCR was performed using Brilliant SYBR Green qPCR master mix (Applied Biosystems, San Francisco, CA, USA) in a Step One Plus Real-Time PCR System (Applied Biosystems, San Francisco, CA, USA). The relative gene transcript expression was assessed using the 2^-ΔΔCt^ method after normalization to expression of GAPDH or 18S rRNA (Qiagen, Valencia, CA, USA). Primer sequences used according to the published literature as follows: IL-23 5’-TCTCCTTCTCCGCTTCAAAATC-3’ (forward) and 5`-GGCGGCTACAGCCACAAA-3`(reverse); for IL-17A 5'- TCCCACGAAATCCAGGATGC -3`(forward) and 5`- GGATGTTCAGGTTGACCATCAC-3' (reverse); for Sdc-1 5`-TACTAATTTGCCCCCTGAAGAT-3`(forward) and 5`-CAAGGTGATATCTTGCAAAGCA-3`(reverse). For DLL4 detection, we employed ABI Master-Mix and the predesigned TaqMan gene expression systems Hs00184092 m1 (DLL4) and Hs99999901_s1 (18S rRNA) (Applied Biosystems, San Francisco, CA, USA).

### Sodium dodecyl sulfate-polyacrylamide gel electrophoresis (SDS-PAGE) and immunoblotting

Briefly, total cell lysates of control and Sdc-1 siRNA transfected SUM-149 cells were prepared as described before [3]. Protein concentration was determined using Bradford assay and 50 ug protein per lane were electrophoresed on 10% SDS-PAGE and electrotransferred into nitrocellulose membrane (Millipore, Germany). After blocking with 5% BSA in tris- buffered saline with 0.1% tween (TBST) for 1 hour the membrane was probed with 2 ug/mL primary antibody against DLL4 (Santa Cruz Biotech, CA, USA) overnight at 4 °C. On the next day, the membrane was incubated with 1 ug/mL anti-mouse secondary antibody conjugated with horseradish peroxidase (Santa Cruz Biotech, CA, USA) for 1 hour at room temperature. The immunoreactivity was visualized by Enhanced Chemiluminescence (ECL) reaction and BioSpectrum 815 Imaging System (Analytik Jena, USA). β-actin (Santa Cruz Biotech) was used as a loading control.

### Statistical analysis

Statistical analysis was performed using IBM SPSS version 18 (Chicago, IL, USA). Fisher’s exact test was used to evaluate differences between variables. Two groups’ comparisons were evaluated for normally distributed data using Student’s *t* test, and for non-normally distributed data using Mann-Whitney U-test. Correlations were conducted using Spearman’s Rank correlation test. Two-tailed *P* values were considered significant at *P* < 0.05. The data were represented as mean ± SEM.

## Results

### Clinical and pathological patient characteristics

We recruited 34 non-IBC and 21 IBC patients and their clinic-pathological criteria are depicted in Table 1. Carcinoma tissue of IBC patients showed a significant increased incidence of ≥4 positive metastatic lymph nodes (*P* = 0.005) as well as a higher lymphovascular invasion (*P* = 0.001) as compared to non-IBC patients.

**Table 1:** Clinical and pathological characteristics of patients.

Group Characteristics	Non-IBC n = 34	IBC n = 21	*P* value
**Age (Years)**			0.7^a^
**Range**	37–78	39–71	
**Mean± SEM**	56.1±1.9	55.1±1.8	
**Tumor size (cm)**			0.05^b^
**≥ 4**	16 (47%)	15 (71.4%)	
**< 4**	18 (53%)	5 (23.8%)	
**NA**	0	1	
**Tumor grade**			0.1^b^
**I**	7 (20.5%)	0 (0%)	
**II**	20 (59%)	15 (71.4%)	
**III**	7 (20.5%)	5 (23.8%)	
**NA**	0	1	
**Lymphovascular invasion**			0.001^b^
**Positive**	5 (14.7%)	12 (57%)	
**Negative**	29 (85.3%)	8 (38%)	
**NA**	0	1	
**No. of metastatic lymph nodes**			0.005^b^
**< 4**	24 (70.6%)	6 (28.6%)	
**≥ 4**	10 (29.4%)	14 (66.6%)	
**NA**	0	1	
**Estrogen receptor (ER) status**			0.5^b^
**Positive**	24 (70.6%)	13 (62%)	
**Negative**	9 (26.5%)	8 (38%)	
**NA**	1	0	
**Progesterone receptor (PR) status**			1.00^b^
**Positive**	20 (59%)	12 (57%)	
**Negative**	13 (38.2%)	9 (43%)	
**NA**	1	0	
**HER2/neu status**			0.4^b^
**Positive**	10 (29.4%)	9 (43%)	
**Negative**	23 (67.6%)	12 (57%)	
**NA**	1	0	

n = number of patients, NA = not available data

Significant *P* values calculated using a. Student’s *t* test or b. Fisher’s exact test

### Low frequency of circulating Th_1_ and Th_2_ CD4^+^ T cell subsets in blood drained from the tumor microenvironment of IBC compared to non-IBC patients

**Fig 1:**
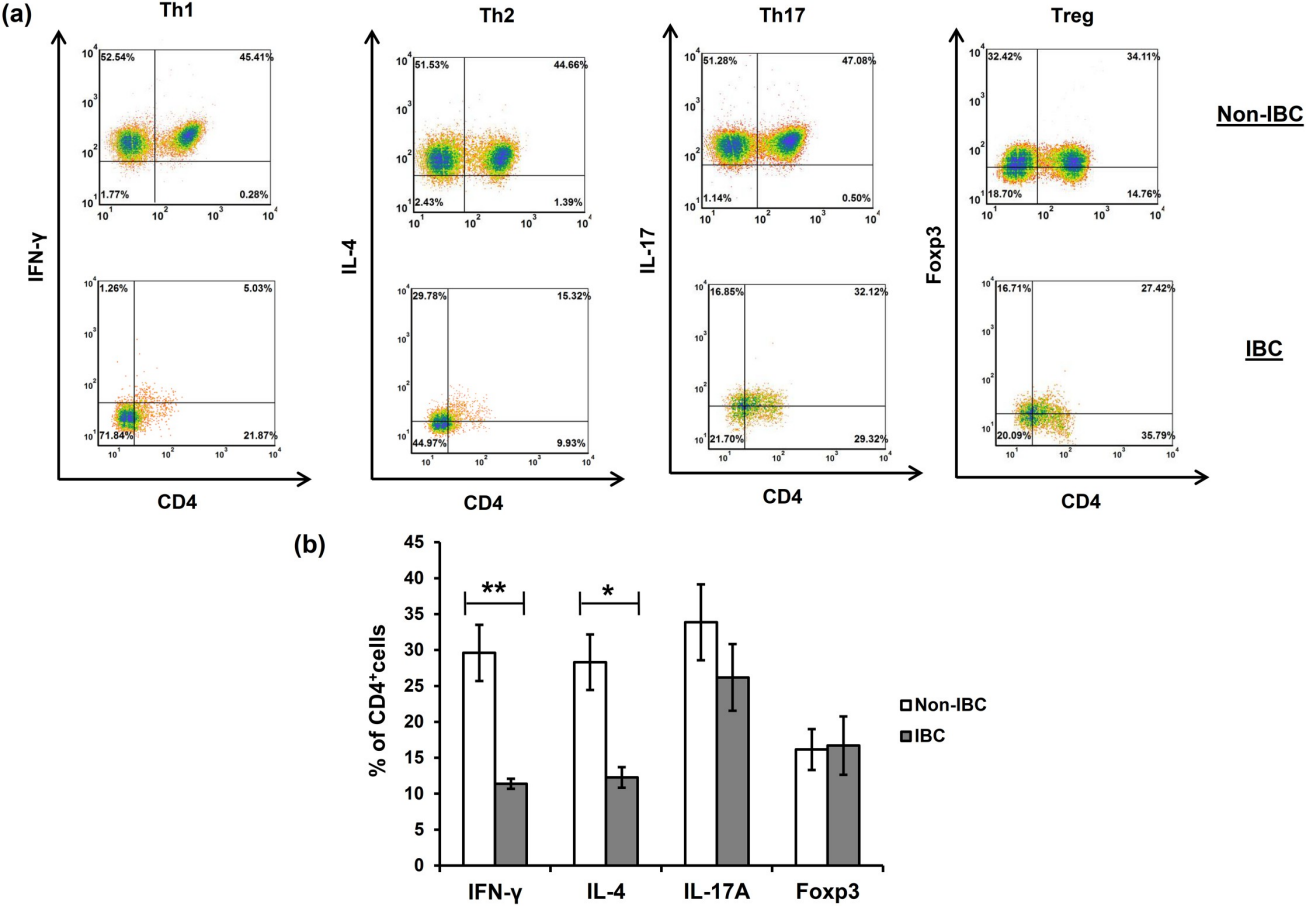
Basal frequencies of T helper subsets isolated from axillary tributaries of non-IBC and IBC patients. Higher proportions of Th_1_ (IFN-γ^+^CD4^+^) and Th_2_ (IL-4^+^CD4^+^) subsets are present in non-IBC than IBC patients. **(a)** Representative flow cytometry dot plots indicating the percentage of cells in each quadrant, and **(b)** Mean percentage ± SEM for non-IBC patients (n = 15), and IBC patients (n = 5). Data shown is representative for a single experiment. * *P* ≤ 0.05, ** *P* ≤ 0.01 as determined by Student’s *t* test.

We have previously detected a significant elevation in the proportion of CD4^+^ T cells drained from the tumor microenvironment of breast cancer patients through axillary tributaries in comparison with peripheral blood [9]. In this study we further characterized CD4^+^ T cell subsets isolated from axillary tributaries of non-IBC and IBC patients, namely Th_1_ (IFN-γ^+^CD4^+^), Th_2_ (IL-4^+^CD4^+^), Th_17_ (IL-17^+^CD4^+^), and T_reg_ (Foxp3^+^CD4^+^) using multicolor flow cytometry. Our data indicate that the frequency of the Th_1_ subset was significantly higher in non-IBC patients by 61.6% than in IBC patients (with an average 29.6 ± 3.9% in non-IBC, and 11.38 ± 0.7% in IBC patients, *P* = 0.01) (Fig 1, Table 2).

**Table 2:** Immunophenotyping of circulating CD4^+^ T helper cells drained from the tumor microenvironment of non-IBC and IBC patients.

T helper phenotype	% of cells in non-IBC (n = 10)	% of cells in IBC (n = 4)	*P* value
Th_1_ (CD4^+^IFN-γ^+^)	29.6 ± 3.9	11.38 ± 0.7	0.01**
Th_2_ (CD4^+^IL-4^+^)	28.29 ± 3.87	12.28 ± 1.43	0.03*
Th_17_ (CD4^+^IL-17^+^)	33.86 ± 5.27	26.18 ± 4.64	0.43
T_reg_ (CD4^+^Foxp3^+^)	16.15 ± 2.84	16.71 ± 4.06	0.92

Values represent mean percentage ± SEM. n = number of patients.

Statistically significance as determined by Student *t* test expressed as * *P* ≤ 0.05, ** *P* ≤ 0.01

Similarly, a significantly increased Th_2_ subset in non-IBC patients by 56.6% compared with IBC was observed (with an average 28.29 ± 3.87% in non-IBC, and 12.28 ± 1.43% in IBC patients, *P* = 0.03) (Fig 1, Table 2). Strikingly, the Th_17_ subset represented the predominant subset among different CD4^+^ T cell subsets, constituting more than 30% of the isolated CD4^+^ T cells in non-IBC (30.37%, with an average 33.86 ± 5.27%, n = 10) and IBC patients (37.82%, with an average 26.18 ± 4.64%, n = 4) (Table 2), although there was no significant difference between non-IBC and IBC patients (Fig 1). Also, the Foxp3^+^ (T_reg_) cell subset ranked as the second highest proportion of CD4^+^ T cells in IBC patients representing 23.1% (with an average 16.71 ± 4.06%, n = 4), whereas it ranked as the lowest proportion of CD4^+^ T cells in non-IBC patients representing 14.56% (with an average 16.15 ± 2.84%, n = 10), although there was no significant difference between non-IBC and IBC patients (Fig 1, Table 2).

### Tumor Sdc-1 silencing significantly favors polarization of Th_1_, Th_17_ and T_reg_ subsets in non-IBC patients

**Fig 2:**
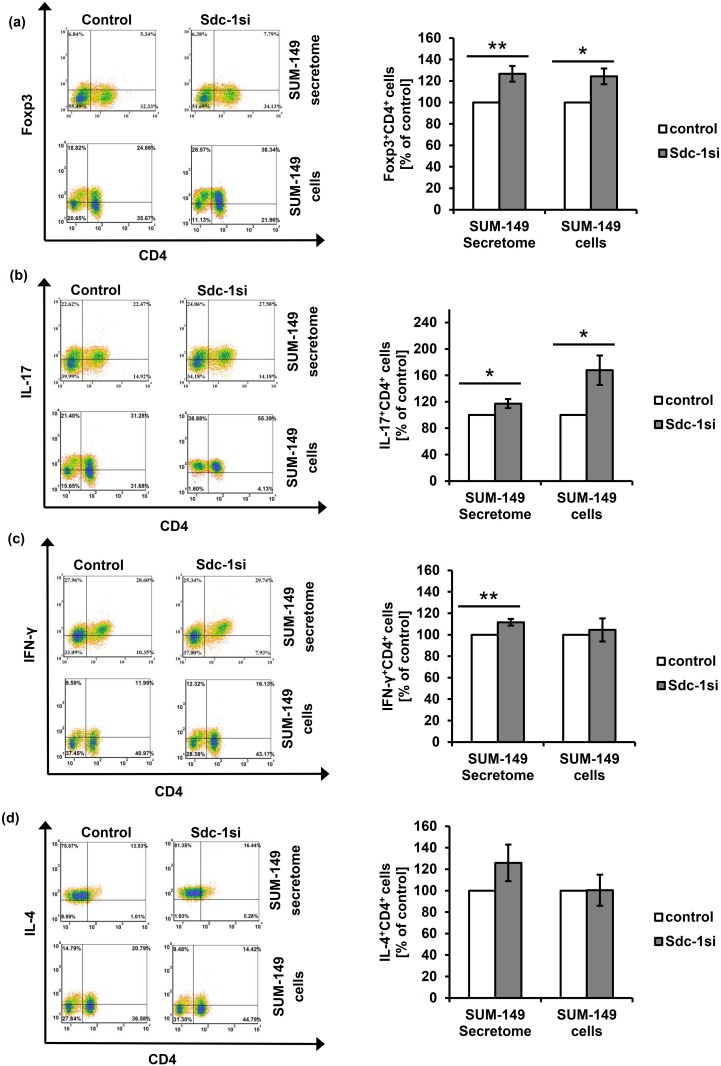
Effect of tumor Sdc-1 silencing on the polarization of T helper subsets of non-IBC patients. Lymphocytes isolated from axillary blood of non-IBC patients were either stimulated by the secretome of control and Sdc-1-silenced SUM-149 cells for 96 h or directly co-cultured with control and Sdc-1-silenced SUM-149 cells for 48 h. Lymphocytes were then stained with labeled antibodies against CD4-FITC, IFN-γ-PE, IL-4-PEcy7, IL-17-PE, and Foxp3-PEcy7. Relative to control cells, both Sdc-1-silenced SUM-149 cells and their secretome enhance **(a)** T_reg_ (Foxp3^+^CD4^+^) and **(b)** Th_17_ (IL-17^+^CD4^+^) subsets, whereas only the secretome of Sdc-1-silenced SUM-149 cells augments **(c)** the Th_1_ (IFN-γ^+^CD4^+^) subset. No effect of Sdc-1 silencing in SUM-149 cells on **(d)** the Th_2_ (IL-4^+^CD4^+^) subset was detected upon stimulation with their secretome or in co-culture. Left panels of **(a-d)** are representative flow cytometric analyses of CD4^+^ T cell subsets. Data shown is representative for a single experiment. Right panels of **(a-d)** show the quantification of CD4^+^ T cell subsets as analyzed by flow cytometry. Data represent mean ± SEM, n = 15 for samples stimulated by the secretome of SUM-149 cells, and n = 5 for samples co-cultured with control and Sdc-1-silenced SUM-149 cells. * *P* ≤ 0.05, ** *P* ≤ 0.01 as determined by Student’s *t* test.

We first confirmed the successful downregulation of Sdc-1 expression by more than 90% at mRNA level and by approximately 65% at the protein level in SUM-149 cells 48h post transfection by qPCR and flow cytometry, respectively (S1 Fig). These cells were used to explore the impact of tumor Sdc-1 silencing on polarization of CD4^+^ T cells isolated from non-IBC (under direct and indirect co-culture conditions) and IBC patients (under indirect co-culture conditions) towards Th_1_, Th_2_, Th_17_ and T_reg_ subsets. Our flow cytometric results revealed that non-IBC CD4^+^ T cells stimulated with the secretome of Sdc-1-silenced SUM-149 cells significantly favored T_reg_ (Foxp3^+^CD4^+^), Th_17_ (IL-17^+^CD4^+^), and Th_1_ (IFN-γ^+^CD4^+^) skewing. The T_reg_ population was altered by 26.7% (with an average 25.3 ± 3.5% in control cells, and 30.8 ± 4.2% upon Sdc-1 silencing, *P* = 0.001, n = 15), the Th_17_ population by 17.2% (with an average 27.4 ± 4.2% in control cells, and 31.9 ± 5.2% upon Sdc-1 silencing, *P* = 0.02, n = 15), and Th_1_ cells by 11.6% (with an average of 37.05 ± 5.07% in control cells and 40.63 ± 5.46% upon Sdc-1 silencing, *P* = 0.001, n = 15) (Fig 2a–2c, Table 3) as compared to control.

**Table 3:** Summary of CD4^+^ T helper subsets of non-IBC and IBC patients upon stimulation with the secretome of control and Sdc-1-silenced SUM-149 cell.

Phenotype	Non-IBC (n = 15)		*P* value	IBC (n = 5)		*P* value
	Control (%)	Sdc-1si (%)		Control (%)	Sdc-1si (%)	
Th_1_	37.05 ± 5.07	40.63 ± 5.45	0.001**	29.04 ± 6.7	30.12 ± 5.8	0.28
Th_2_	25 ± 4.4	29.7 ± 4.9	0.15	35.89 ± 6.8	36.54 ± 5.9	0.29
Th_17_	27.4 ± 4.2	31.9 ± 5.2	0.02*	37.20 ± 7.7	35.47 ± 7.4	0.33
T_reg_	25.3± 3.5	30.8 ± 4.2	0.001**	24.03 ± 4.9	26.56 ± 8	0.95
Th_1_/T_reg_	1.59 ± 0.17	1.65 ± 0.16	0.5	1.57 ± 0.4	2.18 ± 0.8	0.57

Values represent mean percentage ± SEM. n = number of patients.

Statistically significance as determined by Student *t* test expressed as * *P* ≤ 0.05, ** *P* ≤ 0.01

Additionally, in a co-culture of mononuclear cells isolated from non-IBC patients with control and Sdc-1-silenced SUM-149 cells, we observed significantly increased percentages in the T_reg_ (Foxp3^+^CD4^+^) subset by 24.3% (with an average 25.2 ± 4.5% in control cells, and 30.6 ± 4.3% upon Sdc-1 silencing, *P* = 0.02, n = 5) (Fig 2a, Table 4) and in the Th_17_ (IL-17^+^CD4^+^) subset by 67.8% (with an average 26.3 ± 4.2% in control cells, and 41.1 ± 5.4% upon Sdc-1 silencing, *P* = 0.03, n = 5) (Fig 2b, Table 4) in comparison with control. However, we did not observe any significant effect on skewing towards the Th_2_ (IL-4^+^CD4^+^) subset under both conditions and on the Th_1_ (IFN-γ^+^CD4^+^) subset under direct co-culture conditions (Fig 2c and 2d, Table 4).

**Table 4:** Summary of CD4^+^ T helper subsets of normal subjects and non-IBC patients upon direct co-culture with control and Sdc-1-silenced SUM-149 cells.

Phenotype	Normal (n = 5)		*P* value	Non-IBC (n = 5)		*P* value
	Control (%)	Sdc-1si (%)		Control (%)	Sdc-1si (%)	
Th_1_	23.8 ± 3.1	22.31 ± 1.6	1.00	21 ± 3.5	20.4 ± 2.4	0.72
Th_2_	15.54 ± 0.9	12.44 ± 0.7	0.11	14.6 ± 1.5	15.1 ± 3.6	0.98
Th_17_	25.4 ± 3.9	28.32 ± 2.2	0.24	26.3 ± 4.2	41.1 ± 5.4	0.03*
T_reg_	22.61 ± 3.21	28.98 ± 2.56	0.04*	25.2 ± 4.5	30.6 ± 4.3	0.02*
Th_1_/T_reg_	1.1 ± 0.15	0.78 ± 0.04	0.09	0.87 ± 0.1	0.71 ± 0.08	0.23

Values represent mean percentage ± SEM. n = number of patients.

Statistically significance as determined by Student *t* test expressed as * *P* ≤ 0.05

**Fig 3:**
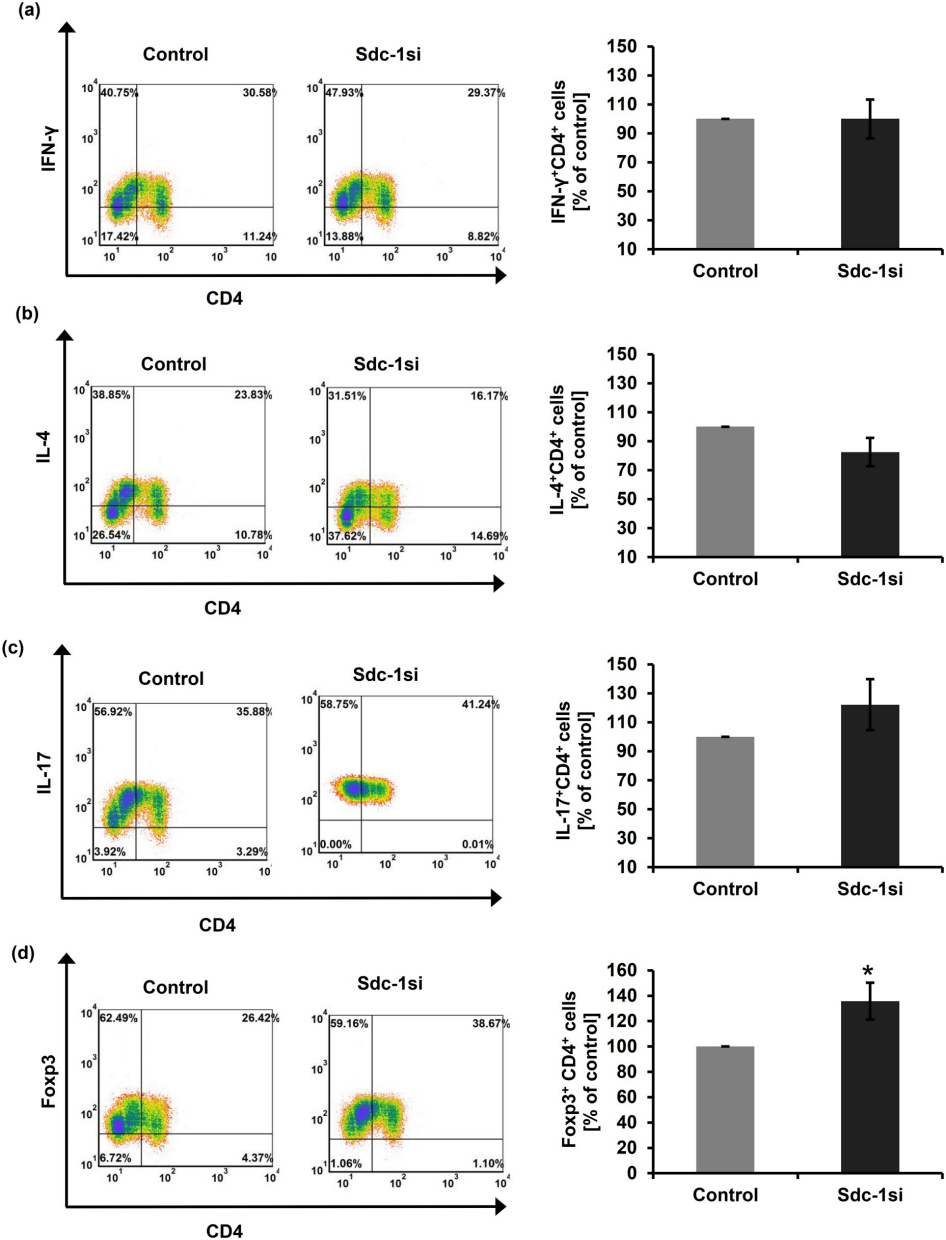
Flow cytometric analysis of normal T helper subsets upon direct co-culture with Sdc-1-silenced SUM-149 cells. PBMCs isolated from normal subjects were co-cultured with Sdc-1-silenced SUM-149 cells for 48 hours. Lymphocytes were then stained with labeled antibodies against CD4-FITC, IFN-γ-PE, IL-4-PEcy7, IL-17-PE, and Foxp3-PEcy7. Relative to control cells, tumor Sdc-1 silencing significantly increased the percentage of **(d)** T_reg_ (Foxp3^+^CD4^+^) subsets while did not change the percentage of the other CD4^+^ T cell subsets **(a-c)**. Left panels of **(a-d)** are representative flow cytometric analyses of CD4^+^ T cell subsets. Data shown is representative for a single experiment. Right panels of **(a-d)** show the quantification of CD4^+^ T cell subsets as analyzed by flow cytometry. Data represent mean ± SEM, n = 5, * *P* ≤ 0.05 as determined by Student’s *t* test.

Further, in a direct co-culture of normal PBMCs with control and Sdc-1-silenced SUM-149 cells, we detected only a significantly increased percentage in the T_reg_ (Foxp3^+^CD4^+^) subset by 35.66% (with an average 22.61 ± 3.21% in control cells, and 28.98 ± 2.56% upon Sdc-1 silencing, *P* = 0.04, n = 5) (Fig 3d, Table 4), whereas there were no significant alterations in the percentage of other subsets as compared to the negative control (Fig 3a–3c, Table 4).

On the contrary, no significant change in the percentages of Th_2_, Th_17_ and T_reg_ subsets of IBC patients was observed when stimulated with the secretome of Sdc-1-silenced SUM-149 cells in comparison to that of control (S2 Fig, Table 3). Only the percentage of the Th_1_ (IFN-γ^+^CD4^+^) subset increased by 9.22% but it did not reach the significance level (*P* = 0.2, n = 5) (S2 Fig, Table 3). Interestingly, the Th_1_/Foxp3^+^ T_reg_ ratio was increased from 1.5 to 2.18 upon stimulation with the secretome of control and Sdc-1-silenced SUM-149 cells (Table 3), suggesting a skewing of CD4^+^ T cell polarization towards the Th_1_ subset as functional effector cells.

### Carcinoma tissues of IBC display an inverse correlation between expression of Sdc-1 and each of IL-4, IL-17, and Foxp3 mRNA levels

**Fig 4:**
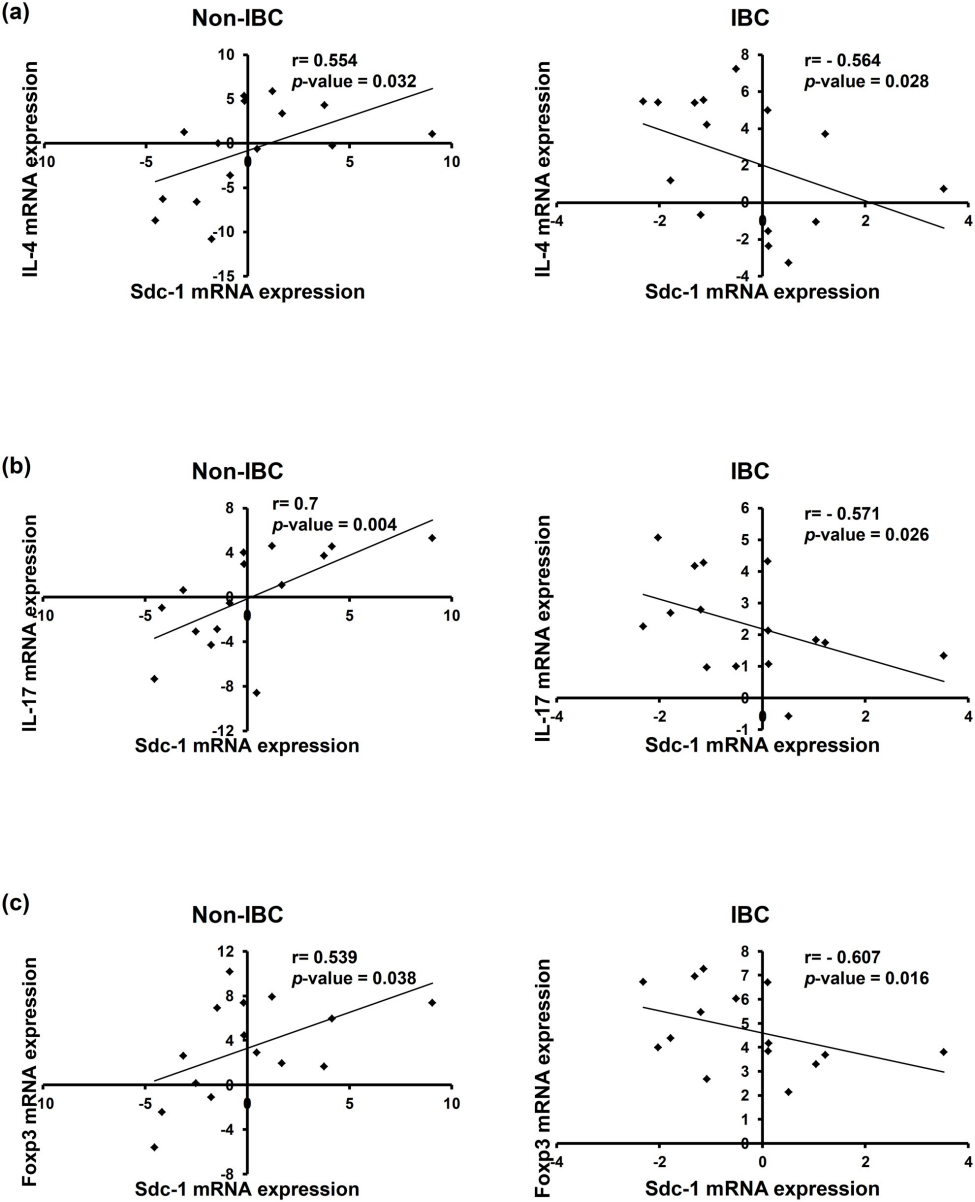
Spearman’s correlation between expression of Sdc-1 and each of IL-4, IL-17, and Foxp3 mRNA levels in carcinoma tissue of non-IBC and IBC patients. Total RNA was extracted from non-IBC and IBC carcinoma tissues collected during surgical operation, reverse transcribed into cDNA, and relative mRNA expression of Sdc-1, IL-4, IL-17, and Foxp3 was determined by qPCR. Spearman`s rank correlation between Sdc-1 and a) IL-4, b) IL-17, and c) Foxp3 mRNA expression in carcinoma tissue of non-IBC (n = 15, positive correlation, left panel) vs. IBC patients (n = 15, negative correlation, right panel). RQ values of mRNA expression are log2-transformed and normalized to values of normal tissues collected during reduction mammoplasty.

We next investigated the clinical relevance of our in vitro data. Therefore, we examined expression of IL-17, Foxp3, IL-4 and Sdc-1 transcript levels in carcinoma tissue of non-IBC and IBC patients and tested whether there is any correlation among them. Interestingly, our qPCR data demonstrate a significantly negative correlation between expression of Sdc-1 and IL-4 (r = - 0.564, *P* = 0.028), IL-17 (r = - 0.571, *P* = 0.026), and Foxp3 (r = - 0.607, *P* = 0.016) in breast carcinoma tissues of IBC patients (Fig 4). In contrast, a significantly positive correlation between expression of Sdc-1 and IL-4 (r = 0.554, *P* = 0.032), IL-17 (r = 0.7, *P* = 0.004), and Foxp3 (r = 0.539, *P* = 0.038) was evident in breast carcinoma tissues of non-IBC (Fig 4). Although no statistical difference for expression of IL-4, IL-17 and Foxp3 mRNA levels in carcinoma tissues of non-IBC and IBC patients was observed, there was a trend for upregulation of IL-4 and downregulation of Foxp3 mRNA levels in carcinoma tissues of IBC in comparison to non-IBC (S3 Fig). This conforms to our in vitro findings and suggests a potential role of Sdc-1 in regulating expression of IL-4, IL-17, and Foxp3 in non-IBC and IBC.

### Sdc-1 knockdown drives Th_17_ skewing possibly via upregulation of IL-23 and DLL4 in SUM-149 cells

**Fig 5:**
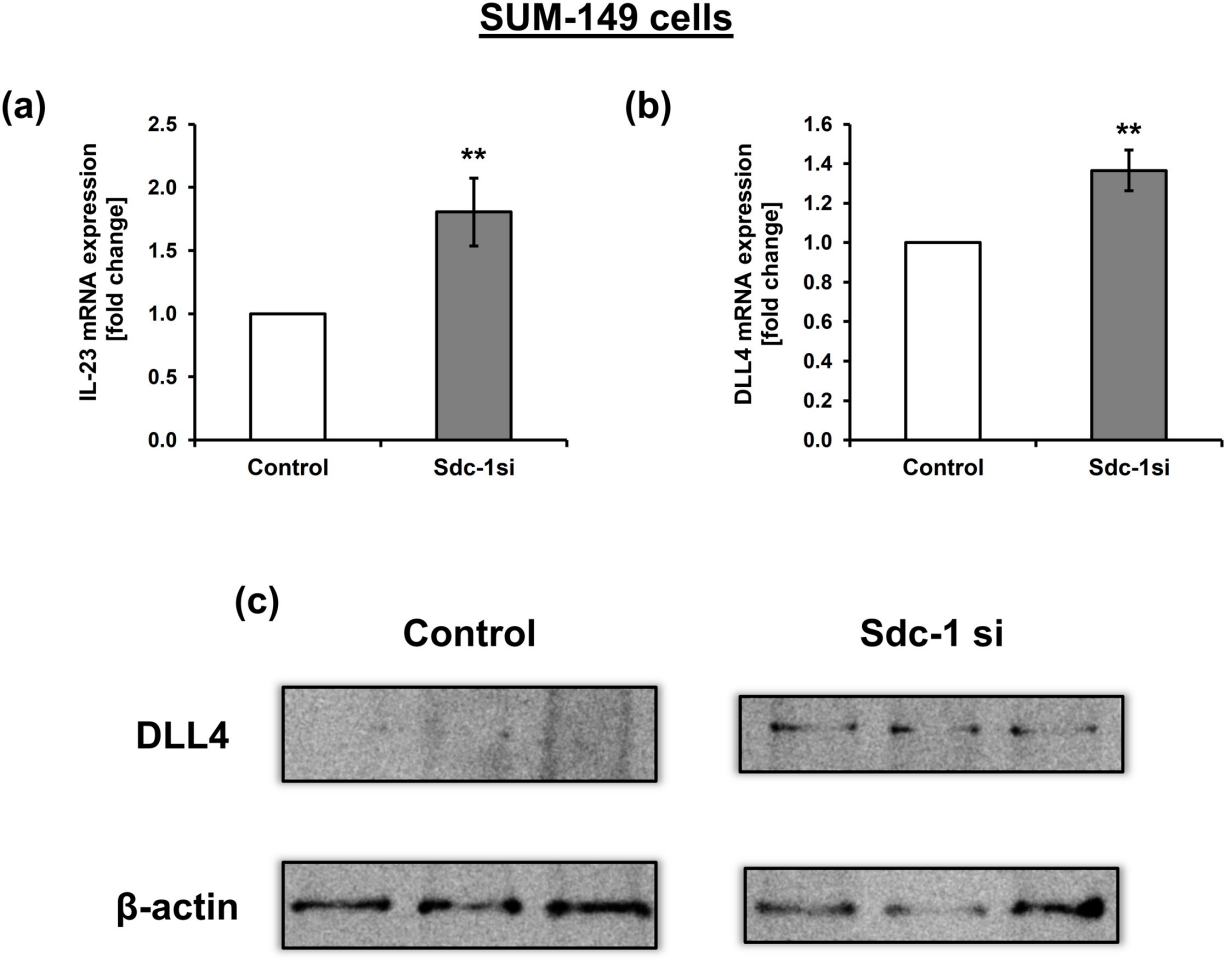
Sdc-1 silencing upregulates IL-23 and DLL4 expression in SUM-149 cells. 48 hours post transfection of SUM-149 with negative control and Sdc-1 siRNA, total RNA was extracted, reverse transcribed into cDNA and relative mRNA expression was quantified by qPCR. mRNA expression of a) IL-23, and b) DLL4 in control and Sdc-1-silenced SUM-149 cells. c) Western blot shows upregulation of DLL4 protein expression in Sdc-1-silenced SUM-149 cells relative to control. Data represent mean ± SEM, n ≥ 3, ** *P* < 0.01 as determined by Student’s *t* test.

We next sought to delineate the potential molecular mechanism(s) exerted by Sdc-1 in regulating Th17 subset. One of the key cytokines that enhance the expansion of Th_17_ cells in inflammatory microenvironment is IL-23 [30]. Thus, we investigated the effect of Sdc-1 silencing on the transcript level of IL-23 in SUM-149 cells. Our qPCR data revealed that Sdc-1 silencing significantly upregulated IL-23 mRNA expression (*P* = 0.01) in comparison to control cells (Fig 5a). Since the Notch ligand delta-like 3 and 4 (DLL3 and 4), provides additional skewing signals to drive the polarization of IL-17 producing Th17 cells [22, 31], we therefore examined whether Sdc-1 silencing might affect the expression of DLL3 and 4 in SUM-149 cells using qPCR and Western blot. Relative to controls, DLL4 was significantly upregulated at the mRNA level in Sdc-1-silenced SUM-149 cells (*P* = 0.0036) (Fig 5b), as well as at the protein level (Fig 5c). However, DLL3 mRNA expression was not altered (data not shown). We further examined expression of TGF-β mRNA (for Th17 and Foxp3 polarization [32]) by qPCR, as well as TLR4 mRNA and protein expression (for Foxp3^+^ T_reg_ polarization [33]) by qPCR and Western blotting, respectively in SUM-149 cells after Sdc-1 silencing. Our data did not reveal any significant difference for their expression upon Sdc-1 suppression (Data not shown).

## Discussion

Due to the unique aggressive nature of IBC, it is speculated to be integrated with a characteristic tumor microenvironment that enhances its aggressive behavior. Mounting evidence pointed to the unfavorable effect of the reciprocal interactions between breast cancer cells and stromal cells nested in the tumor microenvironment, which allow the advance of breast carcinoma phenotype from being in situ to be invasive and spread to lymph nodes and distant tissues [4].

In this study, we extended our previous findings demonstrating increased CD4^+^ T cells drained from tumor microenvironment of breast cancer patients [9] by evaluating the different CD4^+^ T cell subsets isolated from non-IBC and IBC patients. Our data revealed that the Th_17_ (IL-17^+^CD4^+^) subset is the predominant subset among CD4^+^ T cells in both groups; non-IBC and IBC patients. This is consistent with the previous study reported that a high proportion of Th_17_ subset is a common feature between several cancer entities, including breast carcinoma [34]. Furthermore, our results showed that IBC patients are characterized by a high frequency of the T_reg_ (Foxp3^+^CD4^+^) subset representing 23% of total CD4^+^ T cells drained from the tumor microenvironment. On the other hand, in non-IBC patients, we detected a significantly elevated frequency of Th_1_ (IFN-γ^+^CD4^+^) and Th_2_ (IL-4^+^CD4^+^) subsets in comparison with IBC patients. Taken together, we suggest that the suppressed frequency of the Th_1_ (IFN-γ^+^CD4^+^) subset and the elevated T_reg_ (Foxp3^+^CD4^+^) subset in IBC patients may represent a factor that contributes to the aggressive behavior of IBC, based on the fact that Th_1_ subset are typically the effector cells that mediate the main anti-cancer response [11, 35], whereas the elevated proportion of the T_reg_ (Foxp3^+^CD4^+^) subset suppresses the anti-cancer immune response developing immune tolerance of cancer cells, and thus promote progression and aggressiveness of the cancer [36].

Surprisingly, we have detected a significant high frequency of Th_2_ cells in axillary blood of non-IBC patients in comparison with IBC patients. Although there was a high expression of IL-4 mRNA in carcinoma tissues of IBC vs non-IBC, it did not reach the significance level. It has been reported that Th_2_ type tumor microenvironment can promote tumor metastasis [12], and resistance to chemotherapy and radiotherapy [37]. On the contrary, other studies have reported that loss of GATA-3 expression, a master transcriptional regulator of Th_2_ specific cytokines [38], has been associated with aggressive tumor phenotype and worse prognosis in breast cancer patients [39, 40]. Therefore, these conflicting data needs further validation in a large cohort of patients with a determination of IL-4 expression localization in infiltrated Th cells vs. tumor cells.

As Sdc-1 displays the capacity to bind different ligands involving cell surface, matrix proteins, growth factors, cytokines, and chemokines, it emerges as a candidate modulator of inflammatory processes [41]. Sdc-1 expression is upregulated in human breast carcinoma tissue, and its overexpression is correlated with cancer aggressiveness and poor prognosis [42]. Moreover, we have previously demonstrated that relative to non-IBC, carcinoma tissue of IBC confers a higher expression of Sdc-1 mRNA and protein that are associated with the unique cancer stemness phenotype of IBC [3]. Despite the growing evidence connecting chronic inflammation to breast cancer progression [43, 44], to our knowledge this is the first study investigating the impact of tumor Sdc-1 silencing on polarization of T helper cells towards Th_1_, Th_2_, Th_17_, and T_reg_ subsets isolated from non-IBC and IBC breast cancer patients. Our results revealed that tumor Sdc-1 silencing promotes the proportion of Th_1_ (IFN-γ^+^CD4^+^) subset of non-IBC patients when stimulated with the secretome of Sdc-1-silenced SUM-149 cells but not in direct co-culture, as compared to negative control. Moreover, we noted an increased Th_1_/T_reg_ ratio in CD4^+^ T cells isolated from IBC patients upon stimulation with the secretome of Sdc-1-silenced SUM-149 cells in comparison to control, indicating a skewing of the polarization towards the Th_1_ subset. These findings can be explained based on the previous notion that the HS chains of Sdc-1 competitively inhibit IFN-γ binding to its receptor [45, 46], and consequently, inhibit its biologic activity [45, 47]. Further, we previously demonstrated reduced levels of IL-6 in conditioned media of SUM-149 cells upon Sdc-1silencing [3]. IL-6 is a pleiotropic cytokine that is able to interfere with IFN-γR signaling and to inhibit the gene expression of IFN-γ and Th_1_ polarization [48]. Taken together, it seems possible that silencing the expression of Sdc-1 in breast cancer cells could facilitate the binding of IFN-γ to its receptor on T cells, and abrogate the inhibitory effect of IL-6 on IFN-γ/IFN-γR signaling that would lead to an autoregulation of IFN-γ gene expression in T cells and induce Th_1_ polarization. On the contrary, Sdc-1 silencing did not upregulate the percentage of Th_1_ cells under direct co-culture conditions. This could be reasoned to a difference in the subset used, whereby the total axillary mononuclear cells isolated from non-IBC patients, including B cells, T cells, and monocytes, were employed in a direct contact with cancer cells. Under these conditions, monocytes and B cells, the antigen presenting cells (APCs) known to activate T cells, are cancer educated and suppress the effector Th_1_ cells [49]. On the other hand, under indirect stimulation of the isolated axillary T cells with the secretome of control and Sdc-1-silenced SUM-149 cells, the T cells were pre-activated with CD3/CD28 stimulation, which induce Th_1_ phenotype [50]. These results suggest that Sdc-1 can regulate the polarization of Th_1_ subset according to activation status and the cellular components of the tumor milieu.

Apart from Th_1_ polarization, our data revealed that tumor Sdc-1 silencing significantly up-regulated the proportions of the T_reg_ (Foxp3^+^CD4^+^) subset among CD4^+^ T cells of non-IBC patients when stimulated with the secretome of Sdc-1-silenced SUM-149 cells and in co-culture, as well as enhanced the proportion of the T_reg_ (Foxp3^+^CD4^+^) subset among CD4^+^ T cells of normal subjects when co-cultured with Sdc-1-silenced SUM-149 cells, as compared to negative control. Verifying the corresponding in vivo relationship between Sdc-1 and Foxp3 mRNA expression, we found a negative correlation in carcinoma tissue of IBC patients. However, a positive correlation in the carcinoma tissue of non-IBC patients was observed. One of the essential polarizing cytokines of Foxp3 in CD4^+^ T cells is TGF-β [51]. Recent and previous studies demonstrated that Sdc-1 can bind and inhibit TGF-β responsiveness [52–55]. Moreover, it binds and downregulates its activator protein, Thrombospondin-1 (TSP-1) [53, 56]. Additional data demonstrate that Sdc-1 knockdown increases surface expression of αvβ_6_ integrin, activating latent TGF-β complex, and constitutively elevating TGF-β signaling [57]. While TGF-β is critical for polarization of the T_reg_ (Foxp3^+^CD4^+^) subset, IL-6 negatively regulates the generation of T_reg_ cells induced by TGF-β [58]. We have shown that in breast cancer cells specifically IBC, silencing of Sdc-1 drives downregulation of IL-6 and its signaling pathway components [27, 59]. Although TGF-β mRNA expression was not altered after Sdc-1 knockdown, we can speculate that Sdc-1 silencing in SUM-149 cells increases the activation of TGF-β signaling in CD4^+^ T cells via downregulation of IL-6 expression. Consequently, it promotes the expression of Foxp3 within CD4^+^ T cells and upregulates the proportion of the T_reg_ (Foxp3^+^CD4^+^) subset.

Another important finding is that tumor Sdc-1 silencing significantly induced elevated proportion of the Th_17_ (IL-17^+^CD4^+^) subset among CD4^+^ T cells of non-IBC patients when stimulated by the secretome of Sdc-1-silenced SUM-149 cells and under direct co-culture, conditions as compared to negative controls. Interestingly, we have found that Sdc-1 mRNA expression in carcinoma tissue of IBC patients negatively correlated with expression of IL-17, whereas positively correlated in the carcinoma tissue of non-IBC patients. These findings are in line with a previous study that has shown elevated numbers of Th_17_ cells in Sdc-1 knockout CNS during experimental autoimmune encephalomyelitis [60]. Similarly, Sdc-1 knockout mice showed an increase in the frequency of NKT_17_ cells during thymic development [26]. On the contrary, the expression of Sdc-1 is positively correlated with that of IL-17 in nasal epithelial cells, glandular epithelial cells, and inflammatory cells of nasal polyps [61]. Therefore, the conflicting reports in different experimental systems suggest that Sdc-1 may modulate polarization of Th_17_ subset in the context of the pathophysiological state. IL-23 is a key cytokine that plays important roles in the pathogenesis of inflammatory diseases through the activation and expansion of Th_17_ cells [30]. Indeed, our data demonstrated an upregulation of IL-23 mRNA expression in Sdc-1-silenced SUM-149 cells as compared to control. As an additional clue for driving Th_17_ skewing, we detected an upregulation of DLL4 expression at the transcript and protein levels in Sdc-1-silenced SUM-149 cells relative to control cells. DLL4 is a key Notch ligand that engages Notch receptor on T cell surface activating Notch signaling [22, 62]. The gene promotors of Rorc, IL-17, and IL-23-receptor are direct Notch targets [31, 63] and, accordingly, DLL4 provides additional Th_17_ skewing signals through upregulation of RORγt [31]. Taken together, this provides a possible mechanistic explanation for the enhancement of Th_17_ cell expansion via upregulation of IL-23 and DLL4 after suppression of Sdc-1 expression on IBC cells. Given the pro- or anti-tumor functions of Th_17_ cells [64], the exact function of Th_17_ cells upon culturing with Sdc-1-silenced IBC cells should be studied in detail in the future.

Our in vitro data also showed that tumor Sdc-1 silencing did not significantly alter the proportion of the Th_2_ (IL4^+^CD4^+^) subset among CD4^+^ T cells of non-IBC patients under indirect and direct co-culture conditions. This finding may be reasoned to the downregulation of IL-6 produced by Sdc-1-silenced SUM-149 cells, and upregulation of IFN-γ produced by the enhanced Th_1_ subset under indirect co-culture, as the signaling induced by IL-6 can promote the gene expression of IL-4 within CD4^+^ T cells and subsequently upregulate Th_2_ population [65]. Moreover, high levels of IFN-γ can interfere with the expression of the master transcription factor of the Th_2_ population; GATA-3, and inhibit gene expression of Th_2_ cytokines [66]. Analogously, under direct co-culture conditions, Th_2_ polarization was not altered because of upregulation of DLL4 expression upon Sdc-1 suppression in SUM-149 cells, since DLL4 expression limits Th_2_ cytokine production [31].

**Fig 6:**
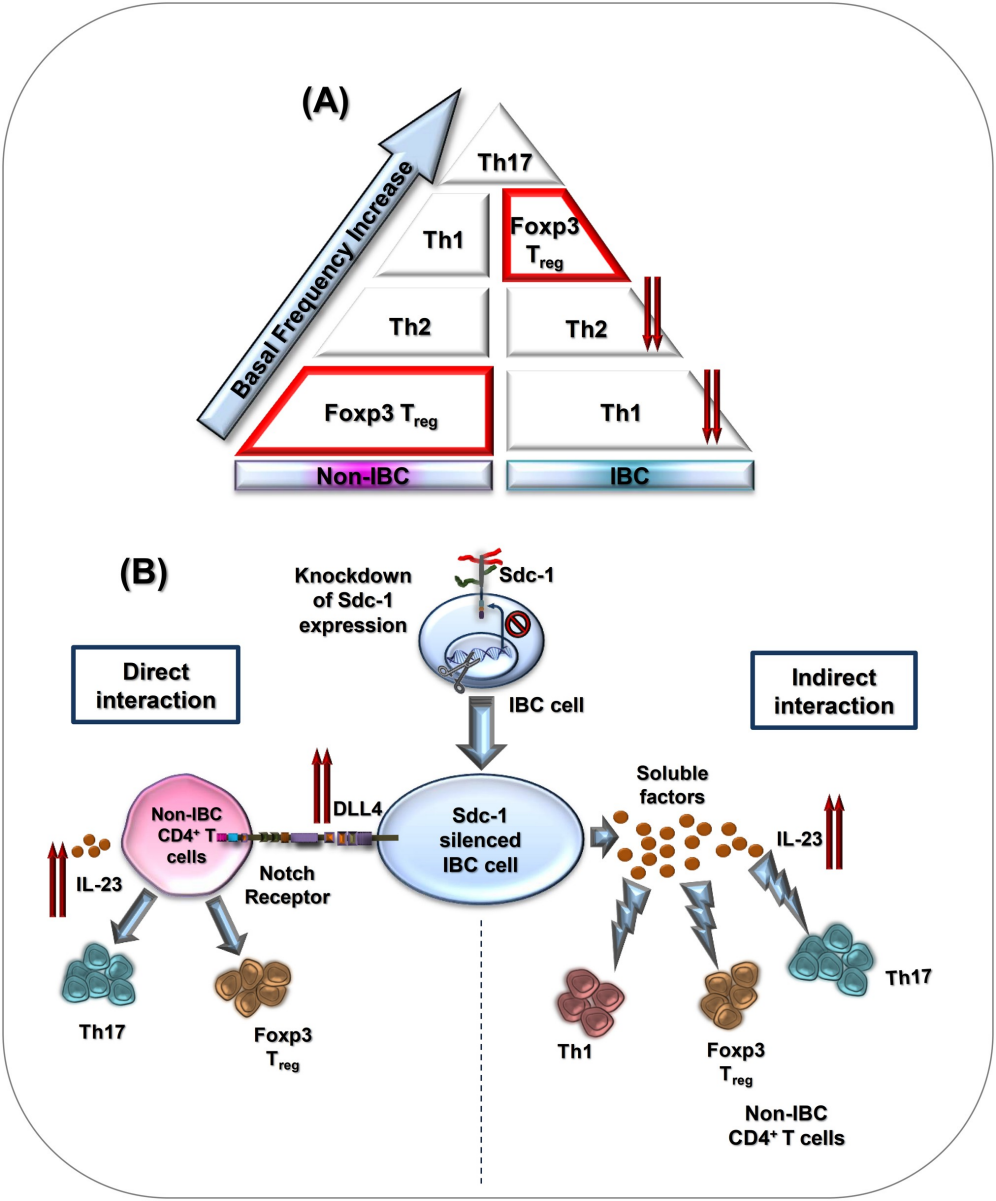
Schematic diagram shows the basal immunophenotyping of axillary Th cells of non-IBC vs. IBC patients, and the immunoregulatory role of tumor Sdc-1 for CD4^+^ Th cell polarization of non-IBC patients. (A) IBC patients are characterized by lower frequencies of circulating Th_1_ and Th_2_ subsets drained from axillary tributaries as compared to non-IBC patients. Relative to other Th subsets, T_reg_ (CD4^+^Foxp3^+^) subset represents the lowest frequency among Th cells of non-IBC patients. (B) Upregulation of IL-23 and DLL4 expression mediated by Sdc-1 silencing in IBC cells may drive Th_17_ polarization under indirect and direct co-culture conditions. In addition, Sdc-1 silencing enhances T_reg_ cell polarization under both indirect and direct co-culture conditions, whereas induces only Th_1_ polarization under indirect co-culture conditions.

In conclusion, our study demonstrates lower frequencies of circulating Th_1_, and Th_2_ subsets in axillary tributaries of IBC than that of non-IBC patients (Fig 6). Moreover, we demonstrate that tumor Sdc-1 silencing enhances the polarization of Th cells under indirect conditions towards Th_1_ and under both co-culture conditions towards Th_17_, and T_reg_ subsets, but not towards Th_2_ subset in non-IBC. However, tumor Sdc-1 silencing does not alter the polarization of Th cells towards a particular subset, but increases only the Th_1_/T_reg_ ratio indicating a skewing of the polarization towards Th_1_ subset in IBC. Moreover, there is a negative correlation between expression of Sdc-1 mRNA and IL-4, IL-17 and Foxp3 mRNA levels in carcinoma tissues of IBC and that correlation was reversed in non-IBC. Mechanistically, Sdc-1 silencing mediates Th_17_ cells expansion possibly via upregulation of IL-23 and DLL4 (Fig 6). Our findings suggest a role for tumor Sdc-1 in modulating the polarization of Th cells within the tumor microenvironment of breast cancer dependent on the type the disease (non-IBC vs. IBC). Further research on large patient collectives is warranted to verify our findings and to clarify the exact functions of different Th subsets induced by Sdc-1 silencing on IBC cells.

## Supporting information

S1 Fig Confirmation of Sdc-1 siRNA transfection in SUM-149 cells. **(a)** Quantitative PCR analysis for mRNA expression of Sdc-1 in control and Sdc-1 siRNA transfected SUM-149 cells. Total cellular RNA was isolated and reverse transcribed into cDNA and gene expression level was measured by qPCR. **(b)** Flow cytometric analysis of Sdc-1 expression in control and Sdc-1 siRNA transfected SUM-149 cells. 500,000 cells were stained for isotype control mouse IgG1-PE and mouse anti-human Sdc-1-PE and the cells were subjected to flow cytometry. Each plot shows mouse IgG-PE control (dark histogram) and Sdc-1-PE-stained cells (white histogram). The median fluorescence intensity (MFI) of events is given for each peak. Data are a single experiment representative of three independent experiments.
(TIF)

S2 Fig Flow cytometric analysis of CD4^+^ T cell subsets of IBC patients upon tumor Sdc-1 silencing. Lymphocytes isolated from axillary blood of IBC patients were stimulated by the secretome of Sdc-1-silenced SUM-149 cells for 96 h. Lymphocytes were then stained with labeled antibodies against CD4-FITC, IFN-γ-PE, IL-4-PEcy7, IL-17-PE, and Foxp3-PEcy7. Relative to control cells, tumor Sdc-1 silencing did not significantly change the percentages of **(a)** Th_1_ (IFN-γ^+^CD4^+^), **(b)** Th_2_ (IL-4^+^CD4^+^), **(c)** Th_17_ (IL-17^+^CD4^+^), and **(d)** T_reg_ (Foxp3^+^CD4^+^) subsets. Left panels of **(a-d)** are representative flow cytometric analysis of CD4^+^ T cell subsets. Data shown is representative for a single experiment. Right panels of **(a-d)** show the quantification of CD4^+^ T cell subsets as analyzed by flow cytometry. Data represent the mean ± SEM, n = 5, statistically significance is considered at *P* ≤ 0.05 as determined by Student’s *t* test.
(TIF)

S3 Fig No significant differences for IL-4, IL-17, and Foxp3 mRNA expression in carcinoma tissue of non-IBC vs. IBC patients. Total RNA was extracted from non-IBC and IBC carcinoma tissue collected during surgical operation, reverse transcribed into cDNA, and relative mRNA expression of a) IL4, b) IL-17, and c) Foxp3 were quantified by qPCR. RQ values of mRNA expression are log2 transformed and normalized to values of normal tissues collected during reduction mammoplasty. n = 15, *P* < 0.05 is considered significant as determined by Mann-Whitney U-test.
(TIF)

## References

[1] Bray F, Ferlay J, Soerjomataram I, Siegel RL, Torre LA, Jemal A. Global cancer statistics 2018: GLOBOCAN estimates of incidence and mortality worldwide for 36 cancers in 185 countries. CA Cancer J Clin. 2018 10.3322/caac.21492 . 30207593

[2] Ibrahim AS, Khaled HM, Mikhail NN, Baraka H, Kamel H. Cancer incidence in egypt: results of the national population-based cancer registry program. Journal of cancer epidemiology. 2014;2014:437971. Epub 2014/10/21. 10.1155/2014/437971 . 25328522 PMC4189936

[3] Ibrahim SA, Gadalla R, El-Ghonaimy EA, Samir O, Mohamed HT, Hassan H, et al Syndecan-1 is a novel molecular marker for triple negative inflammatory breast cancer and modulates the cancer stem cell phenotype via the IL-6/STAT3, Notch and EGFR signaling pathways. Molecular cancer. 2017;16(1):57 Epub 2017/03/09. 10.1186/s12943-017-0621-z . 28270211 PMC5341174

[4] Lim B, Woodward WA, Wang X, Reuben JM, Ueno NT. Inflammatory breast cancer biology: the tumour microenvironment is key. Nature reviews Cancer. 2018;18(8):485–99. Epub 2018/04/29. 10.1038/s41568-018-0010-y . 29703913

[5] Kaminska K, Szczylik C, Bielecka ZF, Bartnik E, Porta C, Lian F, et al The role of the cell-cell interactions in cancer progression. Journal of cellular and molecular medicine. 2015;19(2):283–96. Epub 2015/01/20. 10.1111/jcmm.12408 . 25598217 PMC4407603

[6] Ren Y, Jia HH, Xu YQ, Zhou X, Zhao XH, Wang YF, et al Paracrine and epigenetic control of CAF-induced metastasis: the role of HOTAIR stimulated by TGF-ss1 secretion. Molecular cancer. 2018;17(1):5 Epub 2018/01/13. 10.1186/s12943-018-0758-4 29325547 PMC5765658

[7] Al Absi A, Wurzer H, Guerin C, Hoffmann C, Moreau F, Mao X, et al Actin Cytoskeleton Remodeling Drives Breast Cancer Cell Escape from Natural Killer-Mediated Cytotoxicity. Cancer research. 2018;78(19):5631–43. Epub 2018/08/15. 10.1158/0008-5472.CAN-18-0441 . 30104240

[8] Hollmén M, Roudnicky F, Karaman S, Detmar M. Characterization of macrophage—cancer cell crosstalk in estrogen receptor positive and triple-negative breast cancer. Scientific reports. 2015;5:9188. Epub 2015/03/18. 10.1038/srep09188 PMC4361875 25776849

[9] El-Shinawi M, Abdelwahab SF, Sobhy M, Nouh MA, Sloane BF, Mohamed MM. Capturing and characterizing immune cells from breast tumor microenvironment: an innovative surgical approach. Annals of surgical oncology. 2010;17(10):2677–84. Epub 2010/03/25. 10.1245/s10434-010-1029-9 . 20333554 PMC3402355

[10] Mumm JB, Oft M. Cytokine-based transformation of immune surveillance into tumor-promoting inflammation. Oncogene. 2008;27(45):5913–9. Epub 2008/10/07. 10.1038/onc.2008.275 . 18836472

[11] Ahrends T, Spanjaard A, Pilzecker B, Babala N, Bovens A, Xiao Y, et al CD4(+) T Cell Help Confers a Cytotoxic T Cell Effector Program Including Coinhibitory Receptor Downregulation and Increased Tissue Invasiveness. Immunity. 2017;47(5):848–61.e5. Epub 2017/11/12. 10.1016/j.immuni.2017.10.009 29126798

[12] DeNardo DG, Barreto JB, Andreu P, Vasquez L, Tawfik D, Kolhatkar N, et al CD4(+) T cells regulate pulmonary metastasis of mammary carcinomas by enhancing protumor properties of macrophages. Cancer Cell. 2009;16(2):91–102. 10.1016/j.ccr.2009.06.018 . 19647220 PMC2778576

[13] Cochaud S, Giustiniani J, Thomas C, Laprevotte E, Garbar C, Savoye AM, et al IL-17A is produced by breast cancer TILs and promotes chemoresistance and proliferation through ERK1/2. Scientific reports. 2013;3:3456 Epub 2013/12/10. 10.1038/srep03456 . 24316750 PMC3856404

[14] Welte T, Zhang XH. Interleukin-17 Could Promote Breast Cancer Progression at Several Stages of the Disease. Mediators of inflammation. 2015;2015:804347. Epub 2016/01/20. 10.1155/2015/804347 . 26783383 PMC4691460

[15] Gupta S, Joshi K, Wig JD, Arora SK. Intratumoral FOXP3 expression in infiltrating breast carcinoma: Its association with clinicopathologic parameters and angiogenesis. Acta oncologica (Stockholm, Sweden). 2007;46(6):792–7. Epub 2007/07/27. 10.1080/02841860701233443 . 17653902

[16] Xu L, Xu W, Qiu S, Xiong S. Enrichment of CCR6+Foxp3+ regulatory T cells in the tumor mass correlates with impaired CD8+ T cell function and poor prognosis of breast cancer. Clinical immunology (Orlando, Fla). 2010;135(3):466–75. Epub 2010/02/26. 10.1016/j.clim.2010.01.014 . 20181533

[17] Wan YY. Multi-tasking of helper T cells. Immunology. 2010;130(2):166–71. Epub 2010/06/19. 10.1111/j.1365-2567.2010.03289.x . 20557575 PMC2878461

[18] Gotte M. Syndecans in inflammation. FASEB journal: official publication of the Federation of American Societies for Experimental Biology. 2003;17(6):575–91. Epub 2003/04/01. 10.1096/fj.02-0739rev . 12665470

[19] Baba F, Swartz K, van Buren R, Eickhoff J, Zhang Y, Wolberg W, et al Syndecan-1 and syndecan-4 are overexpressed in an estrogen receptor-negative, highly proliferative breast carcinoma subtype. Breast Cancer Res Treat. 2006;98(1):91–8. 10.1007/s10549-005-9135-2 . 16636895

[20] Nikolova V, Koo CY, Ibrahim SA, Wang Z, Spillmann D, Dreier R, et al Differential roles for membrane-bound and soluble syndecan-1 (CD138) in breast cancer progression. Carcinogenesis. 2009;30(3):397–407. Epub 2009/01/08. 10.1093/carcin/bgp001 . 19126645

[21] Stanley MJ, Stanley MW, Sanderson RD, Zera R. Syndecan-1 expression is induced in the stroma of infiltrating breast carcinoma. American journal of clinical pathology. 1999;112(3):377–83. Epub 1999/09/09. 10.1093/ajcp/112.3.377 . 10478144

[22] Tindemans I, Peeters MJW, Hendriks RW. Notch Signaling in T Helper Cell Subsets: Instructor or Unbiased Amplifier? Frontiers in immunology. 2017;8:419 Epub 2017/05/02. 10.3389/fimmu.2017.00419 . 28458667 PMC5394483

[23] Kharabi Masouleh B, Ten Dam GB, Wild MK, Seelige R, van der Vlag J, Rops AL, et al Role of the heparan sulfate proteoglycan syndecan-1 (CD138) in delayed-type hypersensitivity. Journal of immunology (Baltimore, Md: 1950). 2009;182(8):4985–93. Epub 2009/04/04. 10.4049/jimmunol.0800574 . 19342678

[24] Xu J, Park PW, Kheradmand F, Corry DB. Endogenous attenuation of allergic lung inflammation by syndecan-1. Journal of immunology (Baltimore, Md: 1950). 2005;174(9):5758–65. Epub 2005/04/22. 10.4049/jimmunol.174.9.5758 . 15843578

[25] Xiao J, Angsana J, Wen J, Smith SV, Park PW, Ford ML, et al Syndecan-1 displays a protective role in aortic aneurysm formation by modulating T cell-mediated responses. Arteriosclerosis, thrombosis, and vascular biology. 2012;32(2):386–96. Epub 2011/12/17. 10.1161/ATVBAHA.111.242198 . 22173227 PMC3404811

[26] Dai H, Rahman A, Saxena A, Jaiswal AK, Mohamood A, Ramirez L, et al Syndecan-1 identifies and controls the frequency of IL-17-producing naive natural killer T (NKT17) cells in mice. European journal of immunology. 2015;45(11):3045–51. Epub 2015/08/25. 10.1002/eji.201545532 26300525 PMC4676762

[27] Ibrahim SA, Hassan H, Vilardo L, Kumar SK, Kumar AV, Kelsch R, et al Syndecan-1 (CD138) modulates triple-negative breast cancer stem cell properties via regulation of LRP-6 and IL-6-mediated STAT3 signaling. PloS one. 2013;8(12):e85737 Epub 2014/01/07. 10.1371/journal.pone.0085737 . 24392029 PMC3877388

[28] Ibrahim SA, Yip GW, Stock C, Pan JW, Neubauer C, Poeter M, et al Targeting of syndecan-1 by microRNA miR-10b promotes breast cancer cell motility and invasiveness via a Rho-GTPase- and E-cadherin-dependent mechanism. International journal of cancer. 2012;131(6):E884–96. Epub 2012/05/11. 10.1002/ijc.27629 . 22573479

[29] Dagur PK, McCoy JP Jr. Collection, Storage, and Preparation of Human Blood Cells. Curr Protoc Cytom. 2015;73(1):5 1–16. 10.1002/0471142956.cy0501s73 . 26132177 PMC4524540

[30] Aggarwal S, Ghilardi N, Xie MH, de Sauvage FJ, Gurney AL. Interleukin-23 promotes a distinct CD4 T cell activation state characterized by the production of interleukin-17. The Journal of biological chemistry. 2003;278(3):1910–4. Epub 2002/11/06. 10.1074/jbc.M207577200 . 12417590

[31] Mukherjee S, Schaller MA, Neupane R, Kunkel SL, Lukacs NW. Regulation of T cell activation by Notch ligand, DLL4, promotes IL-17 production and Rorc activation. Journal of immunology (Baltimore, Md: 1950). 2009;182(12):7381–8. 10.4049/jimmunol.0804322 . 19494260 PMC2980695

[32] Zhou L, Lopes JE, Chong MM, Ivanov II, Min R, Victora GD, et al TGF-beta-induced Foxp3 inhibits T(H)17 cell differentiation by antagonizing RORgammat function. Nature. 2008;453(7192):236–40. Epub 2008/03/28. 10.1038/nature06878 . 18368049 PMC2597437

[33] Li J, Yang F, Wei F, Ren X. The role of toll-like receptor 4 in tumor microenvironment. Oncotarget. 2017;8(39):66656–67. Epub 2017/10/17. 10.18632/oncotarget.19105 . 29029545 PMC5630445

[34] Su X, Ye J, Hsueh EC, Zhang Y, Hoft DF, Peng G. Tumor microenvironments direct the recruitment and expansion of human Th17 cells. Journal of immunology (Baltimore, Md: 1950). 2010;184(3):1630–41. Epub 2009/12/23. 10.4049/jimmunol.0902813 . 20026736

[35] Kennedy R, Celis E. Multiple roles for CD4+ T cells in anti-tumor immune responses. Immunological reviews. 2008;222:129–44. Epub 2008/03/28. 10.1111/j.1600-065X.2008.00616.x . 18363998

[36] Tanaka A, Sakaguchi S. Regulatory T cells in cancer immunotherapy. Cell research. 2017;27(1):109–18. Epub 2016/12/21. 10.1038/cr.2016.151 . 27995907 PMC5223231

[37] Shiao SL, Ruffell B, DeNardo DG, Faddegon BA, Park CC, Coussens LM. TH2-Polarized CD4(+) T Cells and Macrophages Limit Efficacy of Radiotherapy. Cancer immunology research. 2015;3(5):518–25. Epub 2015/02/27. 10.1158/2326-6066.CIR-14-0232 . 25716473 PMC4420686

[38] Zhou M, Ouyang W. The function role of GATA-3 in Th1 and Th2 differentiation. Immunologic research. 2003;28(1):25–37. Epub 2003/08/30. 10.1385/IR:28:1:25 . 12947222

[39] Dydensborg AB, Rose AA, Wilson BJ, Grote D, Paquet M, Giguere V, et al GATA3 inhibits breast cancer growth and pulmonary breast cancer metastasis. Oncogene. 2009;28(29):2634–42. Epub 2009/06/02. 10.1038/onc.2009.126 . 19483726

[40] Yoon NK, Maresh EL, Shen D, Elshimali Y, Apple S, Horvath S, et al Higher levels of GATA3 predict better survival in women with breast cancer. Human pathology. 2010;41(12):1794–801. Epub 2010/11/17. 10.1016/j.humpath.2010.06.010 . 21078439 PMC2983489

[41] Stepp MA, Pal-Ghosh S, Tadvalkar G, Pajoohesh-Ganji A. Syndecan-1 and Its Expanding List of Contacts. Advances in wound care. 2015;4(4):235–49. Epub 2015/05/07. 10.1089/wound.2014.0555 . 25945286 PMC4397989

[42] Barbareschi M, Maisonneuve P, Aldovini D, Cangi MG, Pecciarini L, Angelo Mauri F, et al High syndecan-1 expression in breast carcinoma is related to an aggressive phenotype and to poorer prognosis. Cancer. 2003;98(3):474–83. Epub 2003/07/25. 10.1002/cncr.11515 . 12879463

[43] DeNardo DG, Coussens LM. Inflammation and breast cancer. Balancing immune response: crosstalk between adaptive and innate immune cells during breast cancer progression. Breast cancer research: BCR. 2007;9(4):212 Epub 2007/08/21. 10.1186/bcr1746 . 17705880 PMC2206719

[44] Jiang X, Shapiro DJ. The immune system and inflammation in breast cancer. Molecular and cellular endocrinology. 2014;382(1):673–82. Epub 2013/06/25. 10.1016/j.mce.2013.06.003 . 23791814 PMC4919022

[45] Fluhr H, Spratte J, Heidrich S, Ehrhardt J, Steinmuller F, Zygmunt M. Heparin inhibits interferon-gamma signaling in human endometrial stromal cells by interference with the cellular binding of interferon-gamma. Fertility and sterility. 2011;95(4):1272–7. Epub 2010/06/15. 10.1016/j.fertnstert.2010.04.061 20542267

[46] Sadir R, Forest E, Lortat-Jacob H. The heparan sulfate binding sequence of interferon-gamma increased the on rate of the interferon-gamma-interferon-gamma receptor complex formation. The Journal of biological chemistry. 1998;273(18):10919–25. Epub 1998/06/06. 10.1074/jbc.273.18.10919 . 9556569

[47] Daubener W, Nockemann S, Gutsche M, Hadding U. Heparin inhibits the antiparasitic and immune modulatory effects of human recombinant interferon-gamma. European journal of immunology. 1995;25(3):688–92. Epub 1995/03/01. 10.1002/eji.1830250309 . 7705397

[48] Diehl S, Rincon M. The two faces of IL-6 on Th1/Th2 differentiation. Molecular immunology. 2002;39(9):531–6. Epub 2002/11/15. 10.1016/S0161-5890(02)00210-9 . 12431386

[49] Sousa S, Brion R, Lintunen M, Kronqvist P, Sandholm J, Monkkonen J, et al Human breast cancer cells educate macrophages toward the M2 activation status. Breast cancer research: BCR. 2015;17:101 Epub 2015/08/06. 10.1186/s13058-015-0621-0 . 26243145 PMC4531540

[50] Smeets RL, Fleuren WW, He X, Vink PM, Wijnands F, Gorecka M, et al Molecular pathway profiling of T lymphocyte signal transduction pathways; Th1 and Th2 genomic fingerprints are defined by TCR and CD28-mediated signaling. BMC immunology. 2012;13:12 Epub 2012/03/15. 10.1186/1471-2172-13-12 . 22413885 PMC3355027

[51] Fu S, Zhang N, Yopp AC, Chen D, Mao M, Chen D, et al TGF-beta induces Foxp3 + T-regulatory cells from CD4 + CD25—precursors. American journal of transplantation: official journal of the American Society of Transplantation and the American Society of Transplant Surgeons. 2004;4(10):1614–27. Epub 2004/09/16. 10.1111/j.1600-6143.2004.00566.x 15367216

[52] Chen Q, Sivakumar P, Barley C, Peters DM, Gomes RR, Farach-Carson MC, et al Potential role for heparan sulfate proteoglycans in regulation of transforming growth factor-beta (TGF-beta) by modulating assembly of latent TGF-beta-binding protein-1. The Journal of biological chemistry. 2007;282(36):26418–30. Epub 2007/06/21. 10.1074/jbc.M703341200 . 17580303

[53] Regos E, Abdelfattah HH, Reszegi A, Szilak L, Werling K, Szabo G, et al Syndecan-1 inhibits early stages of liver fibrogenesis by interfering with TGFbeta1 action and upregulating MMP14. Matrix biology: journal of the International Society for Matrix Biology. 2018;68–69:474–89. Epub 2018/02/20. 10.1016/j.matbio.2018.02.008 29454902

[54] Rider CC. Heparin/heparan sulphate binding in the TGF-beta cytokine superfamily. Biochemical Society transactions. 2006;34(Pt 3):458–60. Epub 2006/05/20. 10.1042/BST0340458 . 16709187

[55] Rider CC, Mulloy B. Heparin, Heparan Sulphate and the TGF-beta Cytokine Superfamily. Molecules (Basel, Switzerland). 2017;22(5). Epub 2017/05/05. 10.3390/molecules22050713 PMC6154108 28468283

[56] Murphy-Ullrich JE, Poczatek M. Activation of latent TGF-beta by thrombospondin-1: mechanisms and physiology. Cytokine & growth factor reviews. 2000;11(1–2):59–69. Epub 2000/03/10. . 10708953 10.1016/s1359-6101(99)00029-5

[57] Stepp MA, Liu Y, Pal-Ghosh S, Jurjus RA, Tadvalkar G, Sekaran A, et al Reduced migration, altered matrix and enhanced TGFbeta1 signaling are signatures of mouse keratinocytes lacking Sdc1. Journal of cell science. 2007;120(Pt 16):2851–63. Epub 2007/08/02. 10.1242/jcs.03480 . 17666434

[58] Gao Z, Gao Y, Li Z, Chen Z, Lu D, Tsun A, et al Synergy between IL-6 and TGF-β signaling promotes FOXP3 degradation. International journal of clinical and experimental pathology. 2012;5(7):626–33. Epub 2012/09/15. . 22977658 PMC3438759

[59] Hassan H, Greve B, Pavao MS, Kiesel L, Ibrahim SA, Gotte M. Syndecan-1 modulates beta-integrin-dependent and interleukin-6-dependent functions in breast cancer cell adhesion, migration, and resistance to irradiation. The FEBS journal. 2013;280(10):2216–27. Epub 2013/01/08. 10.1111/febs.12111 23289672

[60] Zhang X, Wu C, Song J, Gotte M, Sorokin L. Syndecan-1, a cell surface proteoglycan, negatively regulates initial leukocyte recruitment to the brain across the choroid plexus in murine experimental autoimmune encephalomyelitis. Journal of immunology (Baltimore, Md: 1950). 2013;191(9):4551–61. Epub 2013/10/01. 10.4049/jimmunol.1300931 . 24078687

[61] Gong GQ, Ren FF, Wang YJ, Wan L, Chen S, Yuan J, et al Expression of IL-17 and syndecan-1 in nasal polyps and their correlation with nasal polyps. Journal of Huazhong University of Science and Technology Medical sciences = Hua zhong ke ji da xue xue bao Yi xue Ying De wen ban = Huazhong keji daxue xuebao Yixue Yingdewen ban. 2017;37(3):412–8. Epub 2017/06/07. 10.1007/s11596-017-1749-1 . 28585128

[62] Coutaz M, Hurrell BP, Auderset F, Wang H, Siegert S, Eberl G, et al Notch regulates Th17 differentiation and controls trafficking of IL-17 and metabolic regulators within Th17 cells in a context-dependent manner. Scientific reports. 2016;6:39117. Epub 2016/12/16. 10.1038/srep39117 . 27974744 PMC5156918

[63] Meyer Zu Horste G, Wu C, Wang C, Cong L, Pawlak M, Lee Y, et al RBPJ Controls Development of Pathogenic Th17 Cells by Regulating IL-23 Receptor Expression. Cell reports. 2016;16(2):392–404. Epub 2016/06/28. 10.1016/j.celrep.2016.05.088 . 27346359 PMC4984261

[64] Maniati E, Soper R, Hagemann T. Up for Mischief? IL-17/Th17 in the tumour microenvironment. Oncogene. 2010;29(42):5653–62. Epub 2010/08/24. 10.1038/onc.2010.367 . 20729908 PMC2962667

[65] Heijink IH, Vellenga E, Borger P, Postma DS, de Monchy JG, Kauffman HF. Interleukin-6 promotes the production of interleukin-4 and interleukin-5 by interleukin-2-dependent and -independent mechanisms in freshly isolated human T cells. Immunology. 2002;107(3):316–24. Epub 2002/11/09. 10.1046/j.1365-2567.2002.01501.x . 12423307 PMC1782800

[66] Usui T, Preiss JC, Kanno Y, Yao ZJ, Bream JH, O’Shea JJ, et al T-bet regulates Th1 responses through essential effects on GATA-3 function rather than on IFNG gene acetylation and transcription. The Journal of experimental medicine. 2006;203(3):755–66. Epub 2006/03/08. 10.1084/jem.20052165 . 16520391 PMC2118252

