# Supplementary figures and images for: The immunomodulatory role of tumor Syndecan-1 (CD138) on ex vivo tumor microenvironmental CD4+ T cell polarization in inflammatory and non-inflammatory breast cancer patients
Source: PLoS One. 2019 May 30;14(5):e0217550. doi: 10.1371/journal.pone.0217550 (PMC6542534; doi:10.1371/journal.pone.0217550)

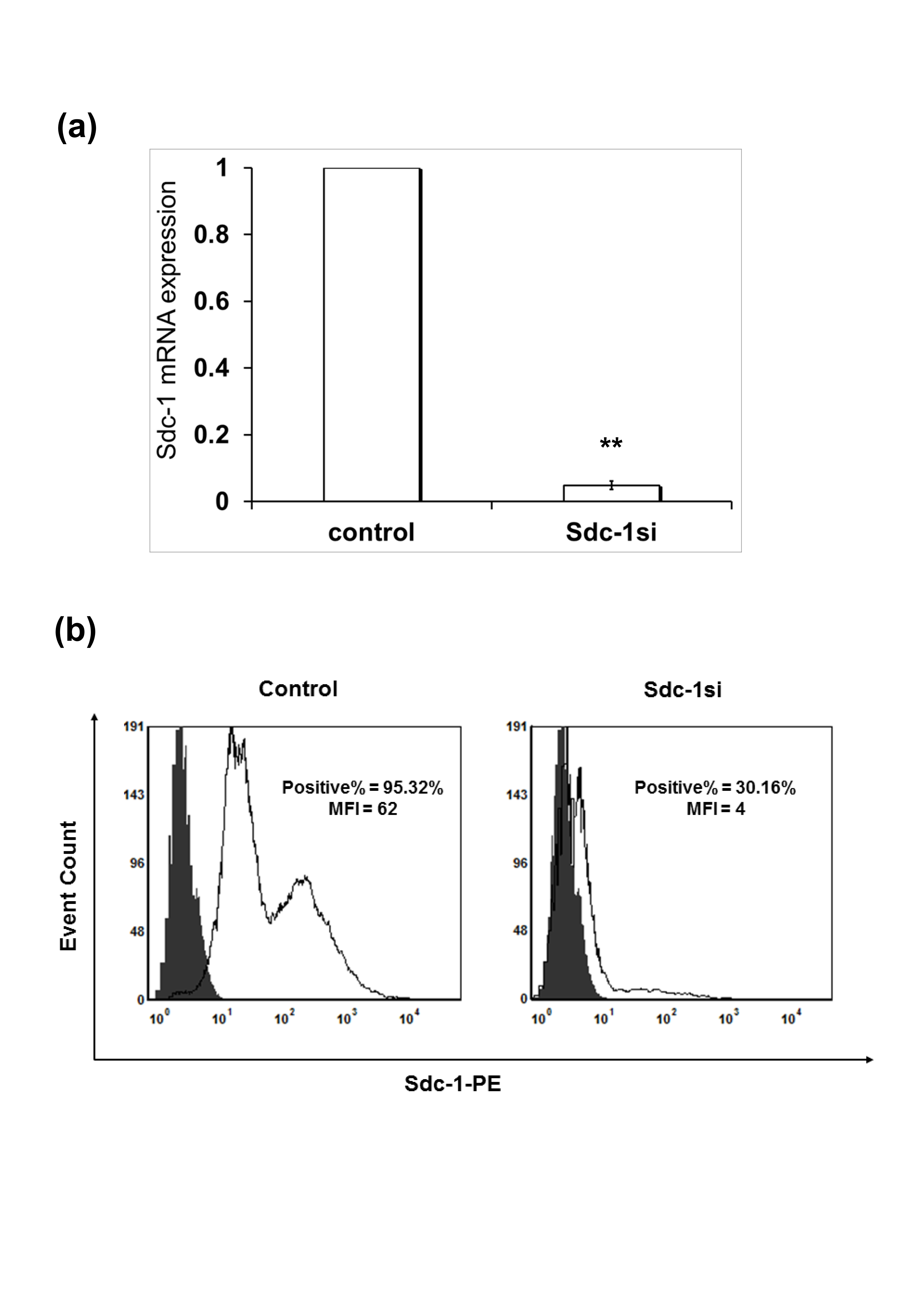

Supplement: S1 Fig — (a) Quantitative PCR analysis for mRNA expression of Sdc-1 in control and Sdc-1 siRNA transfected SUM-149 cells. Total cellular RNA was isolated and reverse transcribed into cDNA and gene expression level was measured by qPCR. (b) Flow cytometric analysis of Sdc-1 expression in control and Sdc-1 siRNA transfected SUM-149 cells. 500,000 cells were stained for isotype control mouse IgG1-PE and mouse anti-human Sdc-1-PE and the cells were subjected to flow cytometry. Each plot shows mouse IgG-PE control (dark histogram) and Sdc-1-PE-stained cells (white histogram). The median fluorescence intensity (MFI) of events is given for each peak. Data are a single experiment representative of three independent experiments. (TIF) [file pone.0217550.s001.tif]

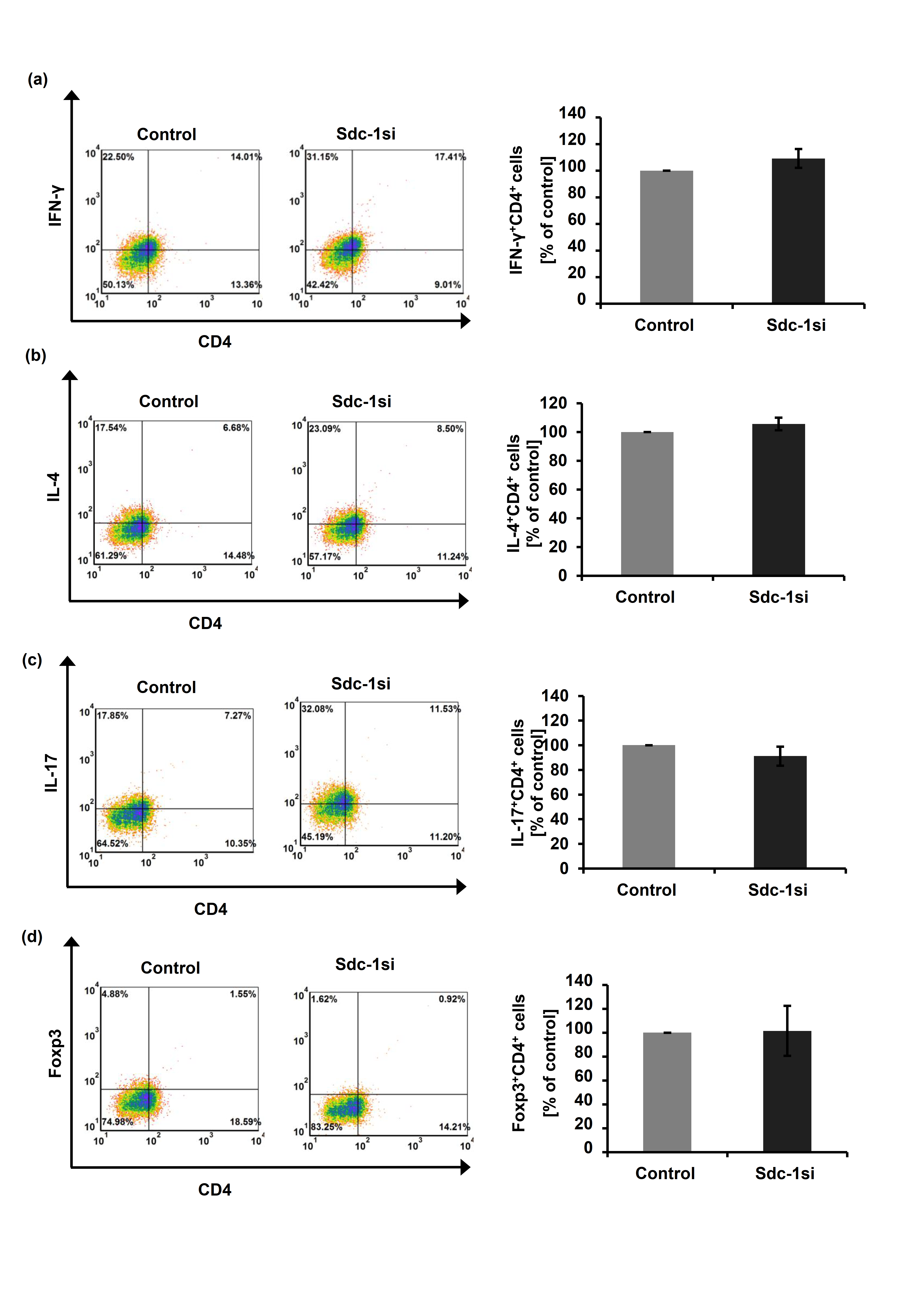

Supplement: S2 Fig — Lymphocytes isolated from axillary blood of IBC patients were stimulated by the secretome of Sdc-1-silenced SUM-149 cells for 96 h. Lymphocytes were then stained with labeled antibodies against CD4-FITC, IFN-γ-PE, IL-4-PEcy7, IL-17-PE, and Foxp3-PEcy7. Relative to control cells, tumor Sdc-1 silencing did not significantly change the percentages of (a) Th1 (IFN-γ+CD4+), (b) Th2 (IL-4+CD4+), (c) Th17 (IL-17+CD4+), and (d) Treg (Foxp3+CD4+) subsets. Left panels of (a-d) are representative flow cytometric analysis of CD4+ T cell subsets. Data shown is representative for a single experiment. Right panels of (a-d) show the quantification of CD4+ T cell subsets as analyzed by flow cytometry. Data represent the mean ± SEM, n = 5, statistically significance is considered at P ≤ 0.05 as determined by Student’s t test. (TIF) [file pone.0217550.s002.tif]

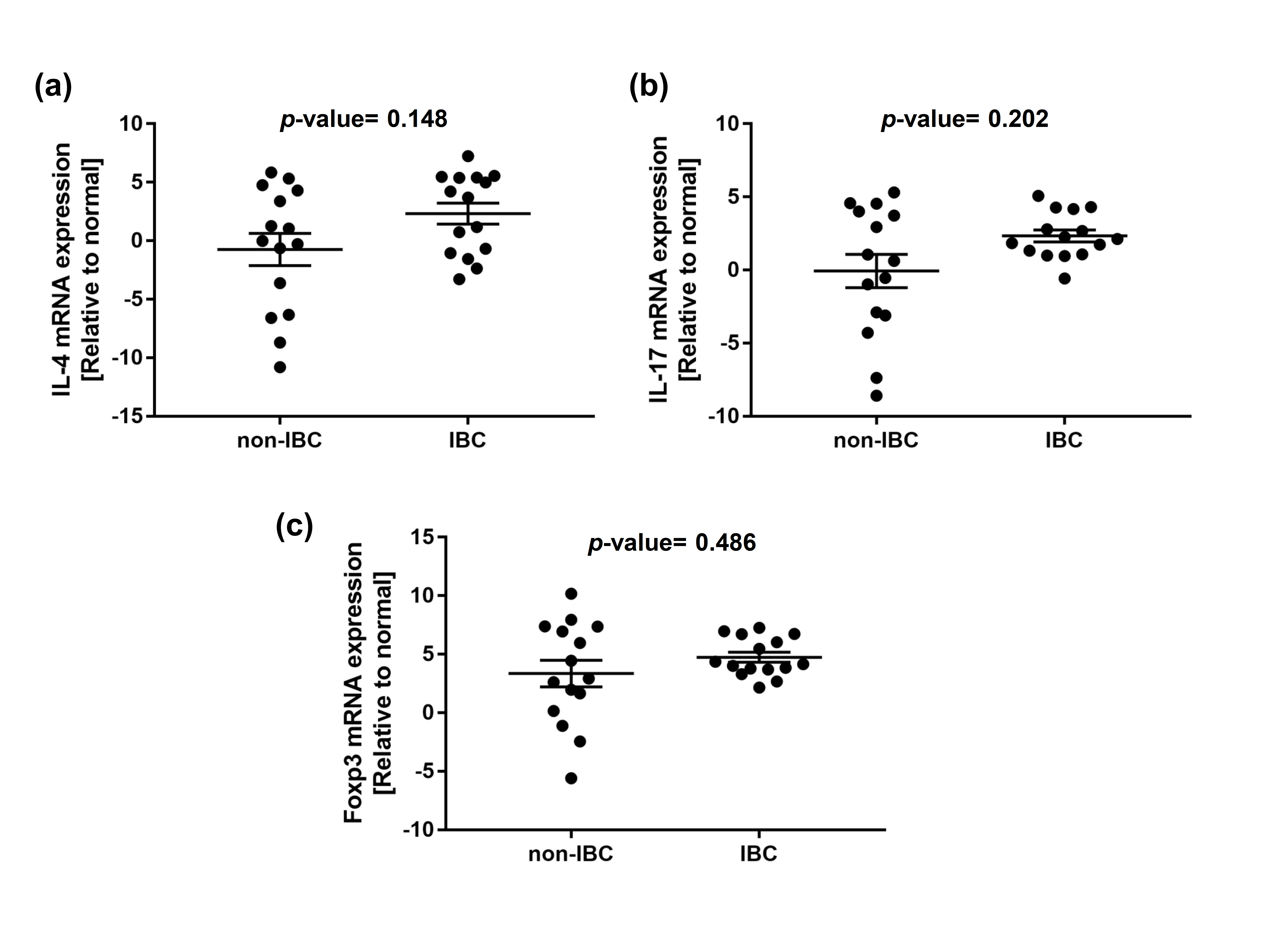

Supplement: S3 Fig — Total RNA was extracted from non-IBC and IBC carcinoma tissue collected during surgical operation, reverse transcribed into cDNA, and relative mRNA expression of a) IL4, b) IL-17, and c) Foxp3 were quantified by qPCR. RQ values of mRNA expression are log2 transformed and normalized to values of normal tissues collected during reduction mammoplasty. n = 15, P < 0.05 is considered significant as determined by Mann-Whitney U-test. (TIF) [file pone.0217550.s003.tif]
